# Association Between Macular Ganglion Cell-Inner Plexiform Layer and Non-Proliferative Retinopathy Without Macular Edema in Type 2 Diabetes via Diabetes Duration and HbA_1_c Link †

**DOI:** 10.3390/biomedicines13020398

**Published:** 2025-02-07

**Authors:** Romano Vrabec, Tomislav Bulum, Spomenka Ljubić, Martina Tomić

**Affiliations:** 1 Department of Ophthalmology, Vuk Vrhovac University Clinic for Diabetes, Endocrinology and Metabolic Diseases, Merkur University Hospital, 10000 Zagreb, Croatia; 2 Department of Diabetes and Endocrinology, Vuk Vrhovac University Clinic for Diabetes, Endocrinology and Metabolic Diseases, Merkur University Hospital, 10000 Zagreb, Croatia; 3 School of Medicine, University of Zagreb, Šalata 3, 10000 Zagreb, Croatia

**Keywords:** ganglion cell-inner plexiform layer, diabetic retinopathy, type 2 diabetes, risk factors

## Abstract

**Background/Objectives:** This study aimed to evaluate the association between the thickness of the macular ganglion cell-inner plexiform layer (GC-IPL), a marker of retinal neurodegeneration, and diabetic retinopathy (DR), a microvasculopathy, in type 2 diabetic patients (T2DM), and to determine the related risk factors. **Methods:** This cross-sectional study included 50 eyes of 25 T2DM with a median age of 64 and a median diabetes duration of 13 years. Complete diabetological, nephrological, and ophthalmological examination was performed, including color fundus photography according to the EURODIAB methodology and optical coherence tomography (OCT) of the macula. Patients with proliferative DR and diabetic macular edema were not included in the study. Data were analyzed using the software package Statistica™ 14.0.1.25 (TIBCO Inc., USA). **Results:** Fifty eyes were divided into two groups: no DR (*n* = 34) and non-proliferative DR (NPDR) (*n* = 16). The NPDR group had longer diabetes duration (*p* = 0.042), higher HbA_1_c (*p* = 0.002), lower HDL cholesterol (*p* = 0.036), and also lower macular GC-IPL thickness (*p* = 0.027) than those without DR. The correlation between DR and GC-IPL was significantly negative (R = −0.319, *p* = 0.024). DR was positively related to diabetes duration (*p* = 0.047) and HbA_1_c (*p* = 0.003), while the relation between GC-IPL and diabetes duration (*p* = 0.042) and HbA_1_c (*p* = 0.043) was negative. Binary logistic regression analysis showed that HbA_1_c (OR = 2.77, *p* = 0.007) and HDL cholesterol (OR = 0.08, *p* = 0.031) were the main predictors for DR, whereas the best model for predicting the GC-IPL thickness (R^2^ = 0.223) obtained from stepwise regression analysis included HDL cholesterol, triglycerides, estimated glomerular filtration rate, and albumin/creatinine ratio. **Conclusions:** The negative correlation between macular GC-IPL and DR in T2DM indicates the coexistence of two parts, neurodegenerative and microvascular, in one diabetic eye complication, linked by the same well-known risk factors: diabetes duration and HbA_1_c.

## 1. Introduction

Diabetic retinopathy (DR) is still the leading cause of preventable visual impairment among adults aged 20–74 years. Most patients with type 1 and type 2 diabetes with disease duration up to 20 years will have DR [1,2]. DR is usually considered, along with diabetic nephropathy, as the most essential microvascular complication in patients with type 2 diabetes (T2DM). Prolonged hyperglycemia in T2DM is associated with chronic retinal microvascular damage, leading to microaneurysms, hemorrhages, hard exudates, and cotton wool spots [3]. Clinical and experimental studies have demonstrated that neurodegenerative changes, especially in the macular ganglion cell-inner plexiform layer (GC-IPL), are also implicated in the development and progression of DR [4,5,6]. In addition, the damage of retinal neurons can be detected even before vascular symptoms [7].

Optical coherence tomography (OCT) is a diagnostic technique for the quantitative structural assessment of GC-IPL [8]. OCT is today routinely used for non-invasive 2-dimensional cross-sectional imaging of the retina [9]. Recent advancements in OCT have enabled the detailed visualization and measurement of individual retinal layers in the macular region [10]. Histological studies have demonstrated age-related loss of retinal ganglion cells, accompanied by a progressive reduction in GC-IPL thickness, as observed through OCT measurements [11]. Systemic and ocular abnormalities can expedite the age-related decline in GC-IPL thickness [12,13]. Diabetes is anticipated to exacerbate the age-related decrease in GC-IPL [14]. The term “neurovascular unit” (NVU) refers to the complex functional interaction and interdependence among neurons, glial cells, the basement membrane, and retinal vascular components [15]. This unit plays a critical role in preserving the integrity of the inner blood–retinal barrier and dynamically regulating blood flow in response to metabolic demands [4].

In patients with T2DM, the normal function of the NVU is compromised, leading to impaired physiological responses early in the disease course, even in those with no or only mild DR [16,17]. The progressive disintegration of the retinal neurovascular unit eventually results in clinically evident retinopathy, characterized by pericyte loss that compromises capillary integrity, weakens the inner blood–retinal barrier, and leads to vascular leakage. Consequently, DR can be regarded not merely as a microvascular disease but also as a sensory neuropathy or neurovascular degeneration [18,19]. The harmful impact of T2DM-associated hyperglycemia on the neural retina has been recognized for over 5 decades [20]. Diabetic retinal neurodegeneration results from several conditions, such as cellular dysfunction, protein kinase C pathway activation, oxidative stress, and increased generation of advanced glycation end products [21].

This study aimed to evaluate the association between the thickness of macular GC-IPL, a marker of retinal neurodegeneration, and DR, a microvasculopathy, in T2DM, and to determine the related risk factors.

## 2. Methods

### 2.1. Study Design and Ethics Statement

This cross-sectional study was performed in Vuk Vrhovac University Clinic for Diabetes, Endocrinology, and Metabolic Diseases, Merkur University Hospital in Zagreb, following the 1964 Helsinki Declaration and its later amendments, and the protocol was approved by the hospital’s ethics committee. The study’s patients received written and oral information about the study and signed written informed consent.

### 2.2. Patients

Twenty-five T2DM consecutively attending Vuk Vrhovac University Clinic for 2 months were included in the study. Type 2 diabetes was defined according to the ADA classification [22]. The study did not include patients with any systemic diseases and disorders (i.e., neurological) and other eye diseases (myopic spherical equivalent < −3 D, hyperopic spherical equivalent > +3 D, mature cataract, uveitis, age-related macular degeneration) that might influence the OCT findings. However, those who met the inclusion criteria were invited to participate in the study. At the inclusion visit, patients signed the informed consent form, their demographic data were assessed, blood and urine samples were collected for laboratory analyses, and complete clinical and ophthalmologic retinal examinations were performed.

### 2.3. Demographic Information and Clinical Factors

The patients’ demographic information included age, gender, and duration of diabetes. Systolic blood pressure (SBP) and diastolic blood pressure (DBP) were measured using an ambulatory mercury sphygmomanometer after a 10 min rest period. The mean value of three measurements was calculated and used for assessment.

### 2.4. Markers of Metabolic Control

Fasting venous blood samples were obtained in the morning after an overnight fast to evaluate metabolic risk factors, which included glycated hemoglobin (HbA_1_c), total cholesterol, HDL cholesterol, LDL cholesterol, and triglycerides. HbA_1_c concentrations were determined using an automated immunoturbidimetric method on a specific analyzer (Cobas Integra 400 Plus, Roche Ltd., Basel, Switzerland). Serum lipid levels were assessed through standard enzymatic procedures on an automated analyzer (Beckman Coulter AU680, Beckman Coulter, Inc., Brea, CA, USA).

### 2.5. Markers of Renal Function

Renal function was assessed by measuring serum creatinine, glomerular filtration rate (GFR), and the albumin/creatinine (A/C) ratio. A fasting blood sample was used to measure serum creatinine using a standard laboratory method. The GFR was calculated using the Chronic Kidney Disease Epidemiology Collaboration (CKD-EPI) formula [23]. A random urine sample was analyzed using turbidimetric immunoassay and photometric methods to determine the A/C ratio.

### 2.6. Ophthalmical Examination of the Retina

The ophthalmological examination of the retina consisted of color fundus photography and OCT of the macula, performed after mydriasis, which was achieved using eye drops with a 0.5% tropicamide. Two color fundus photographs of both eyes were taken using a standard fundus camera, the Visucam NM/FA model manufactured by Zeiss, Jena, Germany based on the EURODIAB retinal photography methodology [24]. Once the images were obtained, two experienced retina specialists independently graded the photographs, assigning a corresponding DR level based on established classification criteria [25]. This dual grading approach helps to enhance reliability and minimize subjective bias in assessing retinal findings. Since there was no case where the experts assigned different DR levels, there was no need for the third grader. OCT of the macula with the evaluation of the macular GC-IPL thickness of both eyes was performed by Spectral Domain OCT (SD-OCT Copernicus REVO, Optopol technology, Zawiercie, Poland). Patients diagnosed with proliferative diabetic retinopathy (PDR), which was confirmed through fundus fluorescence angiography (FFA), and those with diabetic macular edema, characterized by a central foveal thickness (CFT) > 280 μm as measured by the specified SD-OCT device, were excluded from participation in this study [26].

### 2.7. Statistical Analysis

Statistical analysis was performed, and the graphs were created using Statistica™ 14.0.1.25 (TIBCO Software Inc., Palo Alto, CA, USA). The normality of the data distribution was assessed using the Shapiro–Wilk test, while the Levene test was used to evaluate the homogeneity of variance. Descriptive results for continuous variables were presented as means ± SD or medians (min-max), and categorical variables were expressed with numbers. Continuous data differences were analyzed using the *t*-test and the Mann–Whitney test. A non-parametric approach was utilized when the assumption of homogeneity of variance for the tested variables was not met. Categorical data differences were examined using the Chi-square test. The Spearman’s rank correlation test was applied to identify associations between the variables. Binary univariate logistic regression was conducted to determine the main predictors of retinopathy, and stepwise regression analysis was used to find the main predictors of GC-IPL thickness. The threshold for statistical significance in all analyses was established at 0.05.

## 3. Results

### 3.1. Study Population

Fifty eyes of 25 T2DM (15 males/10 females) with a median age of 64 (min 21–max 81) years and a mean diabetes duration of 13 (min 1–max 40) years were included in this cross-sectional study. According to the retinopathy status, they were divided into two groups: no DR (*n* = 17) and non-proliferative DR (NPDR) (*n* = 8). Table 1 presents their demographic data, clinical characteristics, metabolic risk factors, and renal function. The groups did not significantly differ in age (*p* = 0.685), though the NPDR group had more random men than women compared to the no DR group (*p* = 0.016). Patients with NPDR had longer diabetes duration (*p* = 0.042), higher HbA_1_c (*p* = 0.002), and lower HDL cholesterol (*p* = 0.036) than those without DR. No significant difference among groups was found in blood pressure, other metabolic risk factors, and markers of renal function. However, reviewing the medical records, 19 (76%) of the included patients permanently received antihypertensive and 17 (68%) hypolipemic therapy. The macular GC-IPL thickness was significantly lower in patients with NPDR than those without DR (109.5 vs. 114.5 μm, *p* = 0.027) (Table 2).

### 3.2. Correlations and Predictors of Diabetic Retinopathy and GC-IPL Thickness

**Figure 1:**
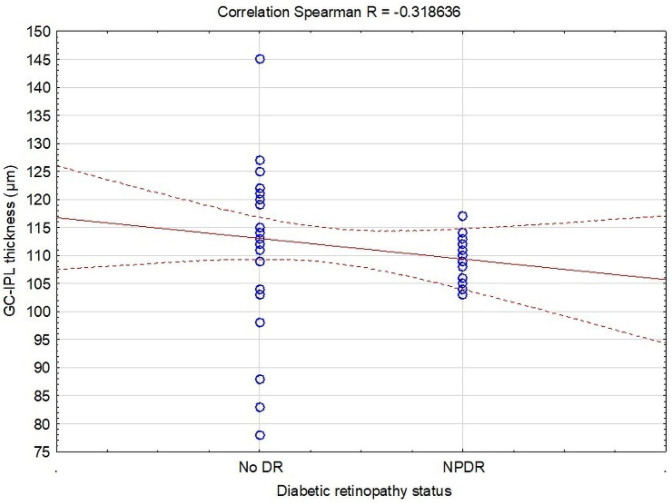
Correlations between diabetic retinopathy and the ganglion cell-inner plexiform layer thickness in patients with type 2 diabetes included in the study. Dotted lines, regression bands and 0.95 confidence interval; line, fitting type; circles, case values.

**Figure 2:**
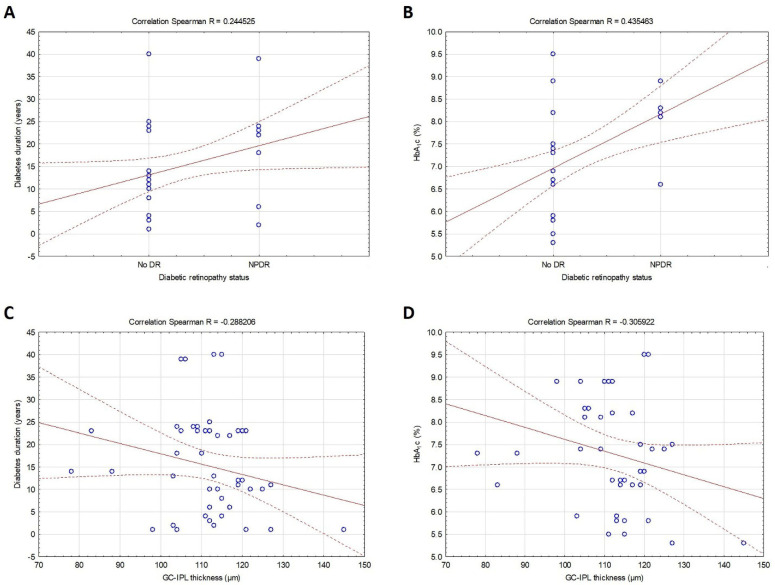
Correlations between diabetic retinopathy, diabetes duration (**A**), and HbA_1_c (**B**), and ganglion cell-inner plexiform layer thickness, diabetes duration (**C**), and HbA_1_c (**D**) in patients with type 2 diabetes included in the study. Dotted lines, regression bands and 0.95 confidence interval; line, fitting type; circles, case values.

The first and most important observation was the negative correlation between DR and macular GC-IPL thickness (R = −0.319, *p* = 0.024) (Table 3, Figure 1). In addition, DR was positively associated with diabetes duration (R = 0.245, *p* = 0.047) and HbA_1_c (R = 0.435, *p* = 0.003), while the relation between GC-IPL thickness and diabetes duration (R = −0.288, *p* = 0.042), and HbA_1_c (R = −0.306, *p* = 0.043) was negative (Table 3, Figure 2).

However, DR was negatively associated with HDL cholesterol (*p* = 0.033), whereas GC-IPL thickness was negatively related to SBP (*p* = 0.016) and A/C ratio (*p* = 0.007) and positively to triglycerides (*p* = 0.032) and eGFR (*p* < 0.001) (Table 3). No significant correlation was observed between DR, GC-IPL, and other analyzed variables (*p* > 0.05).

Binary logistic regression analysis (no DR/NPDR) showed that higher HbA_1_c (OR = 2.77, *p* = 0.007) and lower HDL cholesterol (OR = 0.08, *p* = 0.031) were the main predictors for DR (Table 4), with no significant impact of other analyzed variables. The best model for predicting the GC-IPL thickness (R^2^ = 0.223) obtained from stepwise regression analysis included HDL cholesterol, triglycerides, eGFR, and A/C ratio (Table 5). HDL cholesterol influenced the GC-IPL value with a negative parameter estimate of −10.397611 (*p* = 0.046), while triglycerides with a positive parameter estimate of 7.556523 (*p* = 0.028) relative to a one-unit change of each. Furthermore, other factors related to the GC-IPL value were eGFR with a parameter estimate of 0.311718 (*p* = 0.002) and A/C ratio with a negative parameter estimate of −1.950438 (*p* = 0.039), presenting the negative impact of coexisting diabetic nephropathy (i.e., associated with increased risk of GC-IPL thinning).

## 4. Discussion

The results of our study suggest an association between macular GC-IPL and DR in T2DM, thus indicating the coexistence of two parts, neurodegenerative and microvascular, in one diabetic eye complication, linked by the same well-known risk factors: diabetes duration and HbA_1_c. In addition to microvascular changes in DR, neurodegeneration is thought to have an essential role in the early stages of DR. It represents the death of neuronal cells in the retina and is also related to other eye diseases such as glaucoma and optic neuropathy. At the same time, in DR, neurodegeneration has traditionally been regarded to occur in the late stages of disease rather than as an early manifestation [27]. Neurodegeneration affects the retina on molecular, structural, and functional levels via several factors, such as epithelium-derived factors, corticostatin, and somatostatin, and is present in those without any clinically detectable microvascular changes [28,29,30]. It is important to emphasize that neurodegeneration not only precedes microvascular disease but also plays a significant role in its development and progression [31]. Despite advancements in the treatment of advanced pathology in DR, there is still a need to improve the detection and treatment of early retinal damage.

In our study, macular GC-IPL and DR are linked with well-known risk factors: diabetes duration and HbA_1_c. Retinal neurodegeneration in T2DM is manifested with apoptosis and glial activation. Glial activation negatively affects and damages neuronal cells in the retina via the secretion of toxins, phagocytosis, and finally apoptosis [32]. Several apoptotic markers are increased in retinal ganglion cells in T2DM, including Bad, Bax, Fas, caspase-3, and 9 [33,34]. In those subjects, a reduction in the thickness of the inner retinal layers has been observed with OCT, with minimal association with vascular lesions [6]. Moreover, OCT scans of patients with diabetes at various stages of DR reveal that inner retinal thinning occurs early in the pathology, preceding any detectable vascular signs of DR [29]. Hyperglycemia significantly accelerates neuronal cell death in the retina via increased caspase-3 expression, increased cytochrome c concentration, and reduced content of synaptic proteins in retinal nerve terminals [35,36]. Hyperglycemia is also associated with the greatest level of dysfunction of ganglion cells observed with electroretinogram, and functional abnormalities are present several weeks after the onset of diabetes [37,38]. The multifocal electroretinogram in patients with diabetes observed global retinal dysfunction in the absence of vascular pathology, confirming the importance of neurodegeneration in the development of DR [39]. Hyperglycemia-induced advanced glycation end-products, glutamate excitotoxicity, polyol pathway, activation of protein kinase c, and oxidative stress are implicated not only in the onset and development of DR but also in the apoptosis of retinal neurons [40].

The best model for predicting the GC-IPL thickness in our study, in addition to HDL cholesterol and triglycerides, included eGFR and A/C ratio, presenting the negative impact of coexisting diabetic nephropathy (i.e., associated with increased risk of GC-IPL thinning). Similar to our study, a recently published paper indicated that renal function deterioration was related to thinning of the macular GC-IPL in T2DM [41]. Furthermore, in patients with diabetes, an increase in serum creatinine levels was associated with RNFL thinning, showing also the correlation between renal function and neurodegeneration [42]. A study conducted in the United Kingdom on over 23,000 subjects without neurodegenerative diseases found that kidney disease was associated with a lower baseline GC-IPL thickness [43]. Associations between chronic kidney disease and the thinning of neuroretinal layers were also observed in Asian and White populations [44]. It has been suggested that DR and diabetic nephropathy develop simultaneously and that the severity of DR parallels the presence and severity of diabetic nephropathy because retinal and renal glomerular microvasculature are both highly vascularized organs vulnerable to microvascular damage from systemic diseases [45]. In addition, oxidative stress and chronic inflammation associated with hyperglycemia also cause damage to retinal and renal layers [45]. It is suggested that retinal irregularities, such as GC-IPL thinning, are connected with the development of retinal microvascular damage caused by renal dysfunction [46,47]. Diabetic nephropathy is not only a risk factor for DR but also retinal neurodegeneration.

The results of our study also suggest that GC-IPL thickness and DR are associated with HDL cholesterol. Recently published research that included 10,000 participants found that apart from renal function, lipid metabolism was a major predictor of retinal nerve fiber layer thickness [48]. As in our study, HDL cholesterol was negatively and statistically significantly related to nerve fiber layer thickness, and every 1 mmol/L increase in non-HDL cholesterol was associated with an increase of 0.5 μm in global retinal nerve fiber layer thickness. The retina can quickly uptake cholesterol particles from the circulation, and receptors are expressed on ganglion and glial cells [49]. Treatment with hypolipemic agents can decrease total retinal cholesterol content by 24% [50]. Lipid retention in retinal layers can promote its oxidation and adverse pro-angiogenic and pro-inflammatory effects [51]. On the other hand, relationships between low HDL cholesterol and DR are well documented [52,53]. Serum apolipoprotein A1 (Apo A1), the major protein accounting for about 70% of the total HDL cholesterol, is essential for the reverse-transporting cholesterol from peripheral tissue to the liver [54]. Apo A1 is in a higher concentration expressed within the retinal epithelium in patients with diabetes than in those without it, suggesting its protective effects against lipid deposition and lipo-toxicity [55]. However, systemic inflammation and dyslipidemia appear to be more strongly associated with DR in T2DM compared with patients with type 1 diabetes [56].

The present study and its results have some potential limitations. First, the small number of patients and eyes included in the study is the major limitation, and it does not allow the generalization of these results to T2DM populations. Second, all patients were Caucasian and European subjects, and the findings may not be generalizable to populations of different ethnicities. Third, this was a single hospital-based study, so selection bias is likely. Fourth, we did not include systemic inflammatory markers that might have important effects on GC-IPL thickness and DR associations. Finally, the study design was cross-sectional and did not permit causal conclusions.

## 5. Conclusions

The result of our study suggests a negative correlation between macular GC-IPL and DR in T2DM. Macular GC-IPL and DR are linked with well-known risk factors: diabetes duration and HbA1c. The best model for predicting the GC-IPL thickness in our study included HDL cholesterol, triglycerides, and coexisting diabetic nephropathy. These findings support the role of the retinal vasculature as a biomarker for systemic diseases and the coexistence of two parts, neurodegenerative and microvascular, in one diabetic eye complication.

## Figures and Tables

**Table 1:** Demographic data, clinical characteristics, metabolic risk factors, and renal function of 25 patients with type 2 diabetes divided into two groups according to the retinopathy status.

	No DR (*n* = 17)	NPDR (*n* = 8)	Z ^a^ χ ^b^ t ^c^	*p*-Value
Age (yr) *	64 (21–80)	64 (37–81)	0.406 ^a^	0.685
Gender (m/f) **	8/9	7/1	5.825 ^b^	0.016
Diabetes duration (yr) †	11 (1–40)	22.5 (2–39)	−2.995 ^a^	0.042
SBP (mmHg) *	135 (120–150)	137 (121–150)	−0.432 ^a^	0.666
DBP (mmHg) *	80 (60–95)	82 (72–95)	−0.821 ^a^	0.411
HbA_1_c (%) †	6.96 ± 1.18	8.17 ± 0.80	−3.256 ^c^	0.002
Total cholesterol (mmol/L) †	5.02 ± 1.16	4.38 ± 0.73	1.789 ^c^	0.081
HDL cholesterol (mmol/L) *	1.54 (0.89–1.94)	1.07 (0.90–1.69)	2.095 ^a^	0.036
LDL cholesterol (mmol/L) *	2.93 (1.40–4.70)	2.54 (1.58–2.34)	1.568 ^a^	0.117
Triglycerides (mmol/L)†	1.42 ± 0.47	1.65 ± 0.57	−1.319 ^c^	0.194
Serum creatinine (μmol/L) *	73 (58–99)	72 (68–109)	−0.725 ^a^	0.469
eGFR (mL/min/1.73 m^2^) *	87 (62–110)	92 (62–113)	−0.859 ^a^	0.390
A/C ratio (mg/mmol) *	2.0 (0.2–6.6)	1.3 (0.8–4.8)	0.359 ^a^	0.719

Legend: Values are median (min-max), number, or mean ± SD. Z indicates Mann–Whitney test; χ Chi-square test; t *t*-test; SBP systolic blood pressure; DBP diastolic blood pressure; HbA_1_c glycated hemoglobin; HDL high-density lipoprotein cholesterol; LDL low-density lipoprotein cholesterol; eGFR estimated glomerular filtration rate; A/C albumin-creatinine ratio; DR, diabetic retinopathy; NPDR, non-proliferative diabetic retinopathy; *, median (min-max); **, number; †, mean ± SD.

**Table 2:** Ganglion cell-inner plexiform layer thickness of 50 eyes of 25 patients with type 2 diabetes divided into two groups according to the retinopathy status.

	No DR (*n* = 34)	NPDR (*n* = 16)	Z	*p*-Value
GC-IPL thickness (μm)	114.5 (78–145)	109.5 (103–117)	2.215	0.027

Legend: Values are median (min-max). Z indicates Mann–Whitney test; GC-IPL ganglion cell-inner plexiform layer; DR, diabetic retinopathy; NPDR, non-proliferative diabetic retinopathy.

**Table 3:** Correlations between diabetic retinopathy, ganglion cell-inner plexiform layer thickness, and assessed risk factors in all patients with type 2 diabetes included in the study.

	Diabetic Retinopathy		GC-IPL Thickness	
	Spearman R	*p*-Value	Spearman R	*p*-Value
Diabetic retinopathy	0.000	1.000	−0.319	0.024
GC-IPL thickness	−0.319	0.024	0.000	1.000
Diabetes duration	0.245	0.047	−0.288	0.042
SBP	0.071	0.656	−0.368	0.016
DBP	0.136	0.389	−0.029	0.858
HbA_1_c	0.435	0.003	−0.306	0.043
Total cholesterol	−0.282	0.064	0.088	0.569
HDL cholesterol	−0.322	0.033	−0.193	0.208
LDL cholesterol	−0.241	0.115	0.099	0.524
Triglycerides	0.193	0.209	0.325	0.032
Serum creatinine	0.113	0.466	−0.191	0.215
eGFR	0.140	0.388	0.501	<0.001
A/C ratio	−0.060	0.711	−0.422	0.007

Legend: GC-IPL indicates ganglion cell-inner plexiform layer; SBP systolic blood pressure; DBP diastolic blood pressure; HbA_1_c glycated hemoglobin; HDL high-density lipoprotein cholesterol; LDL low-density lipoprotein cholesterol; eGFR estimated glomerular filtration rate; A/C albumin-creatinine ratio.

**Table 4:** Results of univariate logistic regression analysis for diabetic retinopathy as a dichotomous dependent variable.

Variable	OR (95% CI)	*p*-Value
HbA_1_c	2.77 (1.29–5.94)	0.007
HDL cholesterol	0.08 (0.01–0.85)	0.031

Legend: HbA_1_c indicates glycated hemoglobin; HDL high-density lipoprotein cholesterol.

**Table 5:** Results of stepwise regression analysis for ganglion cell-inner plexiform layer thickness as a dependent variable.

Variable	Estimate	Standard Error	F	*p*-Value	Adjusted R^2^	R^2^
HDL cholesterol	−10.398	5.048	4.24	0.046	0.070	0.223
Triglycerides	7.557	3.309	5.22	0.028	0.089	
eGFR	0.312	0.094	10.89	0.002	0.202	
A/C ratio	−1.950	0.911	4.59	0.039	0.084	

Legend: HDL indicates high-density lipoprotein cholesterol; eGFR estimated glomerular filtration rate; A/C albumin-creatinine ratio.

## Data Availability

The original contributions presented in this study are included in the article. Further inquiries can be directed to the corresponding author.
